# Good old BCG – what a century‐old vaccine can contribute to modern medicine

**DOI:** 10.1111/joim.13195

**Published:** 2020-12-14

**Authors:** C. Locht, M. Lerm

**Affiliations:** ^1^ CIIL‐Centre for Infection and Immunity of Lille Univ. Lille CNRS Inserm CHU Lille Institute Pasteur de Lille Lille France; ^2^ Division of Inflammation and Infection Department of Biomedical and Clinical Sciences Faculty of Medicine and Health Sciences Linköping University Linköping Sweden

**Keywords:** Bacillus Calmette‐Guérin, childhood mortality, covid‐19, melanoma, Type 1 diabetes, tuberculosis

Bacillus Calmette–Guérin (BCG) is derived from *Mycobacterium bovis*, the causative agent of bovine tuberculosis. The bacterium was cultivated between 1908 and 1921, and as demonstrated almost a century later [1], during these years of *in vitro* growth on solid culture media, it lost large regions of chromosomal DNA, some of which encoded important virulence determinants. This loss of virulence made it harmless enough to allow administration of the vaccine to newborns, and it has become the world’s most widely used vaccine. Today, it is recommended by the WHO for the protection of young children against the most severe and deadly forms of tuberculosis [2].

The never‐fading interest in BCG is reflected in PubMed‐listed citations emerging from the simple search ‘BCG’ revealing an accelerating ‘doubling‐rate’ from 220 in 1950 to 400 around the millennial turn to 800 articles in 2020. Just a brief dip in numbers of publications can be noted in the late 50s, probably as a result of the discovery of antibiotics and the premature conclusion that TB was history at that time.

In December 2018, a conference was held at the Institut Pasteur de Lille in Lille, France, the birthplace of BCG. This was the second international workshop dedicated exclusively to BCG and was organized to celebrate the 110^th^ anniversary of the first subculture of *M. bovis*, which finally led to the BCG.

The conference gathered speakers from 17 different countries in Europe, the Americas, Africa, Asia and Australia. All continents were represented, and the presentations covered a wide range of subjects, including the history of BCG, the place of BCG in the fight against mycobacterial diseases, genomics of BCG, next‐generation BCGs, off‐target effects of BCG and the effects of BCG in cancer, autoimmune and inflammatory diseases.

This initially unexpected width of beneficial effects that BCG has unveiled over the years has led to major new discoveries and resulted in treatments against a wide variety of human diseases beyond tuberculosis and leprosy and ranging from cancer to diabetes. The full potential of BCG is only recently being systematically explored, including the possibility that BCG may have a protective effect on coronavirus disease 19 (COVID‐19) [3], which was not heard of at the time of the conference. The underlying mechanisms of this variety of effects are currently an area of intense investigation throughout the world and has greatly contributed to the concept of ‘trained immunity’ [4]. The Lille BCG conference inspired us to prepare a special issue for *Journal of Internal Medicine* by asking some of the speakers at the conference for contributions. In the current issue of *JIM*, we present four articles derived from work presented at the conference and two additional papers.

Early studies from Sweden and France by Naeslund and Albert Calmette himself demonstrated that childhood mortality was reduced after the introduction of BCG for reasons not related to tuberculosis. These findings have later been confirmed in studies from Africa by Stabell‐Benn and colleagues [5], who in this issue of JIM present a meta‐analysis on the correlation between BCG scarring and childhood survival. The study concludes that the presence of a BCG scar is correlated with reduced mortality in children in Guinea–Bissau and suggests that an early time‐point for BCG vaccination (during the first month of life) should be assured in national vaccination programmes. The importance of scar formation is also highlighted by the finding that the formation of a scar, which results from a short period of replication of the BCG at the injection site and the accompanying inflammatory infiltration, is much more strongly associated with the reduction in childhood mortality than just the administration of the vaccine. Finally, the consideration of revaccination of scar‐negative children is discussed as a means to enhance the coverage of effective BCG vaccination in vulnerable populations.

Another unexpected effect of BCG that has been in clinical practice for a very long time is its effect as a cancer therapeutic. It is widely used to treat bladder cancer (the vaccine is repeatedly instilled into the bladder) and has shown to be effective in reducing recurrence of noninvasive bladder cancer. Repeated injections of BCG have long been applied in veterinary science as well, to treat equine sarcoids, ‘white horse melanoma’, and here, Lee and co‐authors elucidate the clinical and molecular aspects of the use of BCG to treat melanoma in humans [6]. The review surveys the existing knowledge of cellular and molecular mechanisms behind the promising effect of BCG as anticancer therapy, including both adaptive immune responses and epigenetic reprogramming associated with trained immunity. The work further summarizes the efficacy and safety of several studies on intralesional BCG therapy for melanoma and compares it with immune checkpoint inhibitor‐treated melanoma, concluding that future studies should explore the possible synergistic effects of these approaches.

Type I diabetes is an autoimmune disorder, in which a dysregulated T‐cell response causes the degradation of insulin‐producing islets of Langerhans in the pancreas. Faustman presents her work on how the metabolic defects observed in immune cells in type I diabetes can be restored in their function through repeated BCG administration [7]. The work expands on the implications of BCG therapy for a number of autoimmune disorders. A mechanism that is discussed for the observed effects involves an increasing number of beneficial regulatory T cells as a result of BCG administration.

A special issue on BCG should of course include work that discusses its use against tuberculosis, and Lindestam Arlehamn and colleagues provide a comprehensive summary of factors known to contribute to the variable antituberculosis efficacy of BCG [8]. Administration routes different from the standard intradermal injection are discussed, including animal studies on intravenous injection and aerosol administration, also providing important information on how the efficacy of BCG could be improved. The work further summarizes the search for immune correlates of BCG protection, which remain enigmatic.

Of course, there are a number of TB vaccine candidates and Harris and co‐workers provide an overview of the highly diverse candidate tuberculosis vaccines in the current pipeline [9]. The work also performs a systematic review of mathematical modelling papers exploring the epidemiological impact of novel tuberculosis vaccines. Modelling studies may inform vaccine trials to optimize delivery strategy and determine population‐level impact and cost‐efficacy. The conclusion of this work is that there is an increasing need for discovery and development of early pipeline candidates and clinical trial capacity to meet the need for effective methods to combat the tuberculosis epidemic.

Finally, in a perspective article, the possible relationship between national BCG vaccination programmes and COVID‐19 is presented [10]. The analysis arrives at the hypothesis that a high population coverage of BCG given to newborns results in heterologous herd immunity, which can protect a population against uncontrolled spread of COVID‐19.

In summary, the century‐old vaccine BCG is still one of the world’s most widely used vaccines, although it is suboptimal for the prevention of pulmonary tuberculosis in adults. Novel tuberculosis vaccines are in development, some of which are based on the original BCG or novel version of BCG‐like vaccines. A major challenge for these vaccine candidates is to show superior protection to BCG in human populations. It will certainly take time before it can be universally replaced by novel approaches to prevent TB. In addition, the increasingly well‐appreciated beneficial nonspecific effects of BCG make it a very valuable tool for public health in general. Therefore, there is no doubt that BCG will continue its journey throughout the 21^st^ century and gradually reveal more and more of its hidden secrets.

## Conflict of interest statement

No conflict of interest was declared.
